# Randomized, open-label, comparative phase IV study on the bioavailability of Ciclosporin Pro (Teva) versus Sandimmun® Optoral (Novartis) under fasting versus fed conditions in patients with stable renal transplants

**DOI:** 10.1186/s12882-019-1340-z

**Published:** 2019-05-14

**Authors:** Anja Gäckler, Sebastian Dolff, Hana Rohn, Johannes Korth, Benjamin Wilde, Ute Eisenberger, Anna Mitchell, Andreas Kribben, Oliver Witzke

**Affiliations:** 1 Department of Nephrology, University Hospital Essen, University Duisburg-Essen, Hufelandstr. 55, 45147 Essen, Germany; 2 Department of Infectious Diseases, University Hospital Essen, University Duisburg-Essen, Hufelandstr. 55, 45147 Essen, Germany

**Keywords:** Cyclosporine, Microemulsion, Gel-based emulsion, Food intake, Bioavailability

## Abstract

**Background:**

The influence of pre- or postprandial administration on pharmacokinetics of cyclosporine is supposed to be less in gel-based formulations than in microemulsions. This study was designed to investigate the influence of a high-fat meal on the pharmacokinetic profile of the two cyclosporine containing formulations Ciclosporin Pro (gel-based emulsion) and Sandimmun®Optoral (microemulsion) in renal transplant recipients.

**Methods:**

A randomized, open-label, repeated-measurement, comparative phase IV trial was conducted with two sequence groups for nutrition condition (fasting→fed, fed→fasting) and two treatment phases (Sandimmun® Optoral → Ciclosporin Pro), each covering both nutrition conditions. Primary pharmacokinetic variable of interest was the reduction of bioavailability due to high-fat food compared to fasting conditions measured by the difference *D* of ln-transformed bioavailability variables (AUC_SS, τ_, C_ss, max_, und C_ss, min_).

**Results:**

A nutrition effect was found for both study medications with respect to the parameters AUC_SS, τ_ and C_SS, max_, but not to C_SS, min_. The reduction of bioavailability caused by high-fat food was not significantly different for Sandimmun®Optoral and Ciclosporin Pro.

**Conclusions:**

An effect of high-fat breakfast prior to the morning dose on AUC_SS, τ_ and C_SS, max_ was found for Sandimmun® Optoral and for Ciclosporin Pro_._ Trough level monitoring did not capture ingestion-related variability. Conversion to Ciclosporin Pro seems to be safe with regard to intra-individual pharmacokinetic variability.

**Trial registration:**

EudraCT No. 2009–011354-18 (29th April 2019)

**Electronic supplementary material:**

The online version of this article (10.1186/s12882-019-1340-z) contains supplementary material, which is available to authorized users.

## Background

Solid organ transplantation has become successful with the development of immunosuppressive therapy. Today, several immunosuppressive substances are available for establishing an individually adapted lifelong immunosuppressive therapy.

Cyclosporine is a powerful immunosuppressant that acts specifically on lymphocytes, mainly T-helper cells. lt inhibits the activation of calcineurin, an important step in the production of lymphokines including interleukin-2, thus resulting in depression of cell-mediated immune response. Cyclosporine has a narrow therapeutic window and is not only used in transplantation, but also for treatment of nephrotic syndrome and various autoimmune diseases. Absorption of conventional formulations of cyclosporine from the gastrointestinal tract is variable and incomplete. An oral microemulsifying formulation, Sandimmun® Optoral, with improved absorption characteristics is available and is more rapidly and completely absorbed, with peak concentrations achieved about 1.5 to 2 h after dose intake (Sandimmun® Optoral Produktmonographie, Novartis Pharma GmbH, Nuremberg 2001).

Ciclosporin Pro formulation is a gel emulsion preconcentrate (GEM) developed by TEVA, registered and marketed in Germany; it is also marketed in Austria as Neoimmune and as Equoral® in different other counties (e.g. Poland, Bulgaria, Latvia, Lithuania, Estonia, Romania, Chile, Mexico, Russia). The formulation is described in publications as GEM 101 [1]. Contrary to the microemulsifying formulation of Sandimmun® Optoral, the addition of small amounts of water to the GEM forms a gel, which can be demonstrated by increasing viscosity of the preparation. This gel with excessive water breaks into particles with different shapes, followed by a rapid decline in viscosity of the created dispersion. The particle diameter is much larger than in microemulsions (100 μm vs. < 150 nm in microemulsions) [1]. It has been postulated that particle size plays a major role in the bioequivalence of cyclosporine [2]. However, a study has suggested that in contrast to previous assumptions, conventional formulations of cyclosporine are quite well absorbed, and that the low bioavailability is due to extensive cytochrome-mediated metabolism in the gut wall. If this was the case, the improved bioavailability seen with the microemulsifying formulation can be presumably less been ascribed to improved absorption than to protection from such metabolism [3]. Moreover, the bioavailability of the GEM formulation has been shown to be bioequivalent to the reference product (cyclosporine microemulsifying formulation) in healthy subjects as well as in renal transplant recipients [1, 3, 4].

The influence of preprandial or postprandial administration on the pharmacokinetics of various cyclosporine formulations has been demonstrated in various studies [5–8]. Two Czech pharmacokinetic studies in healthy volunteers [9, 10] led to generation of the hypothesis that the rate and extent of absorption of the gel-based emulsion Ciclosporin Pro is less influenced by meals than this of the microemulsion.

Strict 12 h dosing intervals are set for patients after transplantation. Administration after fasting is truly only possible in the morning. A formulation which releases cyclosporine independently from or less influenced by food uptake would therefore be preferred. This study was designed to further investigate the influence of a high-fat meal intake compared to fasting conditions on the pharmacokinetic profile of the two cyclosporine containing formulations Ciclosporin Pro (gel-based emulsion) and Sandimmun®Optoral (microemulsion) in stable renal transplant recipients.

## Methods

### Study design

A single-centre randomized, open-label, repeated-measurement, comparative phase IV trial on the bioavailability of Ciclosporin Pro versus Sandimmun® Optoral under fasting versus fed conditions in patients with stable renal transplants was conducted between June 2010 and December 2012.

### Ethics approval

This study was conducted in compliance with Good Clinical Practices (GCP) and the Declaration of Helsinki, and in accordance with applicable legal and regulatory requirements. The study protocol was approved by the ethics commission of the medical faculty of the University Duisburg-Essen and registered in the European Union Clinical Trials Register (EudraCT No. 2009–011354-18).

### Inclusion and exclusion criteria

Detailed inclusion and exclusion criteria can be found in Additional file 1: Figure S1.

### Study procedure

Patients were under stable Sandimmun® Optoral treatment (reference drug) when entering the study. The patients were assigned to the following sequence groups in conformity with two gender-specific randomization lists:

- Group A (fasting → fed):

**Table:** 

PK-profile	1	after 10 h fasting (Sandimmun® Optoral, day 28);
PK-profile	2	after high-fat breakfast (Sandimmun® Optoral, day 29);
PK-profile	3	after 10 h fasting (Ciclosporin Pro, day 57);
PK-profile	4	after high-fat breakfast (Ciclosporin Pro, day 58);

- Group B (fed → fasting):

**Table:** 

PK-profile	1	after high-fat breakfast (Sandimmun® Optoral, day 28)
PK-profile	2	after 10 h fasting (Sandimmun® Optoral, day 29);
PK-profile	3	after high-fat breakfast (Ciclosporin Pro, day 57);
PK-profile	4	after 10 h fasting (Ciclosporin Pro, day 58).

The randomization list was prepared using the software RANCODE 3.6 (IDV Gauting, Germany) by staff of medicomp GmbH who was not involved in the study conduct. The allocation ratio was 1:1. Randomization took place in blocks aiming for a well-balanced group distribution at each recruitment time.

This was an open-label study, thus the investigational team, the patient, and the clinical study monitor knew the sequence group (fasting→fed or fed→fasting) during the study. Nevertheless, data management personnel and the statistician were unaware of the patient’s assignment to Sequence Group A or Sequence Group B during the trial. The laboratory analyzing the blood samples was blinded as well. The database was unblinded on 19 Nov 2013, following completion of data cleaning, data quality control, and database lock. The allocation of patients to the per-protocol set was performed prior to unblinding, i.e. without knowledge of the patient’s sequence group.

Additional file 2: Figure S2 illustrates study procedures.

All patients came to the study center fasting (10 h). Patients either received a standardized normal breakfast or a high-fat breakfast. For high-fat breakfast patients were allowed to choose one of three high-fat breakfasts. Each breakfast consisted of 728–765 kcal of which 55–58% were based on fat. Compositions are shown in detail in Additional file 3: Figure S3. Breakfast was taken at study side under supervision. Patients received standardized meals five hours (lunch) and ten hours (dinner) after morning dose administration. Patients who took the medication under fasting conditions received a standardized breakfast 1 h after morning dose administration. The evening dose was also taken under control of a nurse or medical doctor.

### Adherence

Adherence concerning fasting condition and complete breakfast intake was assessed at the time of each PK profile including the timing of start of breakfast relative to the intake of the study medication. Breakfasts and study medications were taken at study side under supervision. For profiles after high-fat breakfast the time between intake of the morning dose and start of breakfast was planned to be between − 50 and − 10 min (boundaries included), which corresponds to 30 ± 20 min. For profiles after 10 h fasting the desired value was at least 40 min, which corresponds to 60–20 min.

Four pharmacokinetic profiles were captured at day 28 and day 29 (Sandimmun® Optoral) and day 57 and day 58 (Ciclosporin Pro). These pharmacokinetic profiles consisted of plasma concentrations for cyclosporine during τ = 12 h observation starting at morning medication intake and were summarized to the following analysis variables:

- AUC_SS, τ_ area under the cyclosporine-concentration-time curve during one dosing interval τ at steady state
- C_SS, max_ maximal cyclosporine concentration during one dosing interval τ at steady state
- C_SS, min_ minimal cyclosporine concentration during one dosing interval τ at steady state.

Primary pharmacokinetic variable of interest was the reduction of bioavailability due to high-fat food compared to fasting conditions measured by the difference *D* of the natural logarithm (ln)-transformed bioavailability variables (AUC_SS, τ_, C_ss, max_, und C_ss, min_). The primary objective was to examine differences in the nutrition-dependent reduction of bioavailability between Ciclosporin Pro and Sandimmun® Optoral.

The two secondary pharmacokinetic variables used to examine nutrition effects on the bioavailability of Ciclosporin Pro and Sandimmun® Optoral as well as bioequivalence included:

- T_SS, max_ time of maximal cyclosporine concentration during one dosing interval at steady state
- PTF% peak-trough-fluctuation at steady state [%].

### Study medication

Both cyclosporine A formulations, Sandimmun® Optoral and Ciclosporin Pro are approved drugs against rejection following allogenic organ transplantation. In healthy subjects as well as in renal transplant patients bioequivalence of Ciclosporin Pro could be demonstrated in comparison to the originator product Sandimmun® Optoral [4]. The current study concentrates on the aspect of oral nutrition on pharmacokinetic profiles. The study was not primarily designed to evaluate the bioequivalence of two cyclosporine formulations. The exploration of bioequivalence in the current study was performed in a biased subset of patients who had stable C0 levels during the treatment phase, rather than in a randomly selected patient cohort.

Since this was an open-label study with all patients receiving the same medication in identical sequence order, and both medications, test drug and reference drug, have a marketing authorization in Germany, commercially available medication was used for the study. Teva GmbH provided the quantity of Ciclosporin Pro required for the clinical trial. Study medication was dispensed to the patients according to the treatment phase.

### Dosing

Study medication dosage was individualized for each patient.

Dosing was guided by cyclosporine A blood concentrations (C0 level). Intended drug levels depend on concomitant medication against graft rejection and metabolization which shows high inter-individual differences [11]. Therefore, dosing varied between 0.6 and 2.4 mg/kg body weight.

Patients were to receive the same dose (milligram:milligram) of the study medication as the cyclosporine dose the patient had been taken for the past 2 months. Dosage was planned to remain the same again when switching study medication on day 30. To ensure patient safety, cyclosporine blood concentrations (C0 levels) were controlled 7 days after study start (Visit 2) and 7 days after switch of medication from Sandimmun® Optoral to Ciclosporin Pro on day 36 (Visit 5). The medication dose may have been adjusted after these visits if necessary. In case of a dose adjustment, the patient should have come to another control visit 7 days later.

The individual dose of trial medication had to be taken twice daily, at 8.00 o’clock in the morning and at 20.00 o’clock in the evening.

### Concomitant medication

Concomitant medication was recorded in every study visit (see Additional file 2: Figure S2). A potentially relevant protocol violation was the use of medication that might have had an effect on the outcome measures. This could have included but was not limited to the use of other calcineurin inhibitors like tacrolimus and the concomitant use of commercially available formulations of cyclosporine. The administered pre-study and concomitant medication were medically reviewed with a particular focus on medication which was initiated and/or for which the dose was changed less than 5 days (which corresponds to about ten times the half life of cyclosporine) prior to a PK profile. During this review it was decided whether individual violations were minor or major. Medications that were stopped at least 5 days before PK profile 1, started after end of study, or started at least 5 days before PK profile 1 and stopped at or after last visit date were not reviewed as these were not considered candidates for major violations.

Concomitant medication is listed in detail in Additional file 4: Figure S4.

### Cyclosporine a assay

Cyclosporine A was measured by the antibody-conjugated magnetic immunoassay (ACMIA) method [12] (Siemens Healthcare Diagnostics Inc., Newark, USA): patient whole blood in EDTA is lysed and present cyclosporine A conjugated with a monoclonal mouse antibody is sequestered by β-galactosidase. Free antibody-enzyme conjugates are separated by added magnetic particles coated with cyclosporine A. The remaining supernatant containing the antibody-enzyme complex is then mixed with the substrate and β-galactosidase catalyses the hydrolysis of chlorphenol red β-galatopyranoside (CPRG, yellow) to chlorphenol red (red). Photometry is performed at 577 nm (700 nm). The assay ranges from 25 to 500 ng/ml with a functional sensitivity (at 20% coefficient of variation) of 30 ng/ml. For further details on precision, accuracy, and specificity see manufacturer information [13].

### Data acquisition

The full analysis set (FAS) included all randomized patients who received at least one dose of study medication. The per-protocol analysis set (PP) included all patients from the FAS for whom the following criteria were met:

- All four pharmacokinetic profiles evaluable
- all of the major inclusion criteria, none of the major exclusion criteria fulfilled
- absence of relevant protocol violations
- absence of diarrhoea or vomiting at the day of each PK-profile
- sufficient compliance concerning the application of the study medication and concerning the breakfast intake
- absence of dosage adaptations between the pharmacokinetic profiles.

Safety analysis was performed by documentation of adverse events, performance of blood pressure and heart rate measurements, 12-lead ECG, laboratory assessments (creatinine, creatinine clearance, haematology, urine analysis) and cyclosporine C0-levels for monitoring the therapy.

The contract research organization medicomp GmbH (Munich, Germany), who was not involved in the study conduct, was responsible for monitoring, data management, and statistical analysis (data safety and monitoring board, DSMB). Monitoring checked case report forms at time of completion. Quality control included initiation visit and regular monitoring visits for check of completeness, formal and medical plausibility and consistency of the data recorded in case report forms as well as compliance with the protocol including source documentation verification. 100% source data verification was done for 100% of patients and was documented on respective forms. After source data verification, the completed case report forms and other patient documents were collected by the monitor and were forwarded to data management where monitored data were entered and underwent additionally completeness, consistency and plausibility checks.

DSMB advised sponsor with regard to drug safety. Sponsor was allowed to disclose patient data for drug safety reasons.

### Sample size calculation

There were two studies with healthy male volunteers values (Equoral® 100 mg vs. Neoral® 100 mg) which provides estimates for C_max_- and AUC-values under fasting conditions [9] and under fed conditions [10]. The values for C_max_ and AUC from these two studies were used to estimate the difference D of ln-transformed values of bioavailability under fed conditions to bioavailability under fasting conditions (Ciclosporin Pro: D_Cmax_ = − 0.098 and D_AUC_ = − 0.042; Sandimmun Optoral: D_Cmax_ = − 0.330 and D_AUC_ = − 0.218). Estimates for the variances of ln-transformed values were derived using:

- the empirical coefficients of variation of the non-transformed values per treatment:
  - ○ Study under fasting conditions:
    - AUC: CV%_Ciclosporin, fasting_ = 36.79% and CV%_Sandimmun, fasting_ = 50.38%;
    - C_max_: CV%_Ciclosporin, fasting_ = 27.58% and CV%_Sandimmun, fasting_ = 18.08%
  - ○ Study under fed conditions:
    - AUC: CV%_Ciclosporin, fed_ = 24.22% and CV%_Sandimmun, fed_ = 21.44%;
    - C_max_: CV%_Ciclosporin, fed_ = 32.99% and CV%_Sandimmun, fed_ = 24.99%
- different assumptions for within patient correlations.

Sample size calculations were performed using nQuery Advisor 6.0 for a paired t-test, a one-sided α-level of 2.5% and a power of 80%. Calculations based on two studies with healthy male volunteers led to the conclusion that a sample size of 30 evaluable patients, i.e. 30 patients with 4 evaluable pharmacokinetic profiles, should be planned for this study.

### Statistical analysis

The statistical analysis was performed using the software package SAS version 9.3 (SAS Institute Inc., Cary, NC 27513, USA). Primary variables were checked for normal distribution by Kolmogorov-Smirnov test. Statistical analysis was performed by using paired, one-sided t-tests. A one-sided t-test (left-sided) was chosen, as it was assumed that high-fat food reduces the bioavailability of Ciclosporin Pro as well as of Sandimmun® Optoral, and that an increase of bioavailability is neither observed for Ciclosporin Pro or Sandimmun® Optoral, respectively. The two sequence groups were pooled for analysis of primary endpoints. To control for multiplicity in the analysis of primary endpoints hierarchical testing was applied. An overall significance level of 2.5% for primary pharmacokinetic endpoints and 5% for secondary pharmacokinetic endpoints was used. To explore the robustness of results, two analyses of variance (ANOVA) were calculated for each pharmacokinetic parameter of primary interest by using a mixed model with a two-sided significance level of 5%, respectively.

## Results

### Demographic data and baseline characteristics

31 patients were randomized in this single-centre study. All 31 patients received at least one dosage of study medication and were included in the full analysis set (Group A: fasting→fed: 15; Group B: fed→fasting: 16). Demographic data and baseline anamnestic characteristics for the FAS are presented in Table 1. All patients had undergone one kidney transplantation.

**Table 1:** Baseline characteristics (FAS). The sequence groups did not show any relevant differences in terms of gender, age, height, or weight in the FAS or the PP analysis set

		fasting→fed	fed→fasting	Total
		(*n* = 15)	(*n* = 16)	(*n* = 31)
Gender				
Male	n (%)	10 (66.7%)	10 (62.5%)	20 (64.5%)
Female	n (%)	5 (33.3%)	6 (37.5%)	11 (35.5%)
Race		15 (100%)	16 (100%)	31 (100%)
Caucasian	n (%)			
Age [years]	Mean (SD)	48.5 (12.4)	49.9 (14.0)	49.3 (13.1)
	Range	19–67	22–77	19–77
Weight [kg]	Mean (SD)	77.8 (14.3)	78.8 (11.3)	78.3 (12.6)
	Range	55–102	59–102	55–102
Height [cm]	Mean (SD)	174.3 (11.6)	173.9 (14.6)	174.1 (13.0)
	Range	160–200	143–200	143–200
BMI [kg/m^2^]	Mean (SD)	25.5 (3.4)	26.3 (4.0)	25.9 (3.7)
	Range	21.3–30.2	19.8–32.2	19.8–32.2
Smoking habits		9 (60.0%)	13 (81.3%)	22 (71.0%)
Non-smoker	n (%)			
Ex-smoker	n (%)	3 (20.0%)	–	3 (9.7%)
Smoker	n (%)	1 (6.7%)	3 (18.8%)	4 (12.9%)
Unknown	n (%)	2 (13.3%)	–	2 (6.5%)
Time since last renal transplantation [years]	Median (Range)	6.0 (2–28)	6.5 (1–24)	6.0 (1–28)
Number of previous rejection episodes				
0	n (%)	13 (86.7%)	11 (68.8%)	24 (77.4%)
1	n (%)	1 (6.7%)	5 (31.3%)	6 (19.4%)
2	n (%)	1 (6.7%)	–	1 (3.2%)
Time since end of last rejection episode [months]	Median (Range)	36.1 (33–39)	23.7 (6–63)	36.1 (6–63)
		[n = 2]	[*n* = 4]^a^	[*n* = 6]^a^
Duration of last rejection episode [days]	Median (Range)	3.5 (3–4)	4.0 (3–7)	4.0 (3–7)
		[n = 2]	[n = 4]^a^	[n = 6]^a^
Last daily ciclosporin dose applied before baseline [mg/kg/day]	Mean (SD)	2.43 (0.707)	2.31 (0.560)	2.37 (0.628)

^a^ For one patient only year was given for start and end date of last rejection episode (1989). As a result, time since end and duration of last rejection were not assessable for this patient

Overall, there were no clinically relevant differences between sequence groups with respect to the anamnestic characteristics presented in Table 1.

Kidney function was stable in all patients with a mean baseline serum creatinine of 1.82 mg/dl (min. 1.07 mg/dl; max. 2.61 mg/dl) and unchanged during study duration as was eGFR (MDRD) which was gathered at baseline, day 29 (PK 2) and day 58 (PK 4) (Table 2).

**Table 2:** eGFR (MDRD). eGFR was gathered at indicated time points

eGFR (MDRD) [ml/min/1.73 m^2^]	A: fasting→fed	B: fed→fasting	Total
Baseline	38.8 ± 8.8	38.7 ± 11.0	38.7 ± 9.7
Day 29 (PK2)	38.7 ± 10.9	43.5 ± 9.1	41.2 ± 10.0
Day 58 (PK4)	39.8 ± 8.7	40.4 ± 11.4	40.1 ± 10.1

### Concomitant medication

Use of prohibited concomitant therapy was regarded as a major violation in one patient, who reported concomitant use of commercially available formulations of cyclosporine and was therefore excluded from the PP analysis set (see Additional file 5: Figure S5).

### Dosing

The mean initial daily dose of Sandimmun® Optoral was 2.4 mg/kg/day, the mean initial daily dose of Ciclosporin Pro was 2.2 mg/kg/day. Five (23.8%) of the 21 PP patients had at least one change in daily dose (mg) of Sandimmun® Optoral. Three (14.3%) of the 21 PP patients had at least one change in daily dose (mg) of Ciclosporin Pro. None of the study patients had a change in daily dose (mg) at the time of switch from Sandimmun® Optoral to Ciclosporin Pro. Both PK-profiles for the Sandimmun® Optoral and Ciclosporin Pro groups respectively were performed with the same (morning) dose in all 21 PP patients.

### Adherence

All patients were adherent concerning fasting conditions and complete breakfast intake on the day of each PK profile.

Deviations from boundaries found in three patients were classified as major protocol violations and patients were excluded from the per protocol analysis set (see Additional file 5: Figure S5).

### Exclusion from PP analysis set

Ten of the 31 FAS patients (32.3%) were excluded from the PP analysis set. Four patients discontinued the study prematurely. One patient terminated the study due to “patient’s request to withdraw”, three patients were discontinued due to “intolerable adverse events” (pancreatitis, severe leg pain, relapse of ulcerative colitis). Relation to the study medication was medically extremely unlikely for all observed intolerable adverse events, but prohibited completion of the remaining scheduled visits. Furthermore, 6 more patients were excluded from the PP set due to reasons summarized in Additional file 5: Figure S5. Therefore, the PP analysis comprised 21 patients treated with study medication (Group A: fasting→fed: 11 patients; Group B: fed→fasting: 10 patients). In the PP set all patients completed the study entirely in each sequence group. Results of the pharmacokinetic study are reported for the PP set only. It was planned to enroll sufficient patients so that 30 patients would be available with four evaluable pharmacokinetic profiles. However, the study was stopped prematurely after 30 month and the enrolment of 31 patients due to recruitment difficulties within a reasonable time frame. This reduced the power of statistical testing.

#### Descriptive statistics for the primary pharmacokinetic parameters and evaluation of differences

**Fig. 1:**
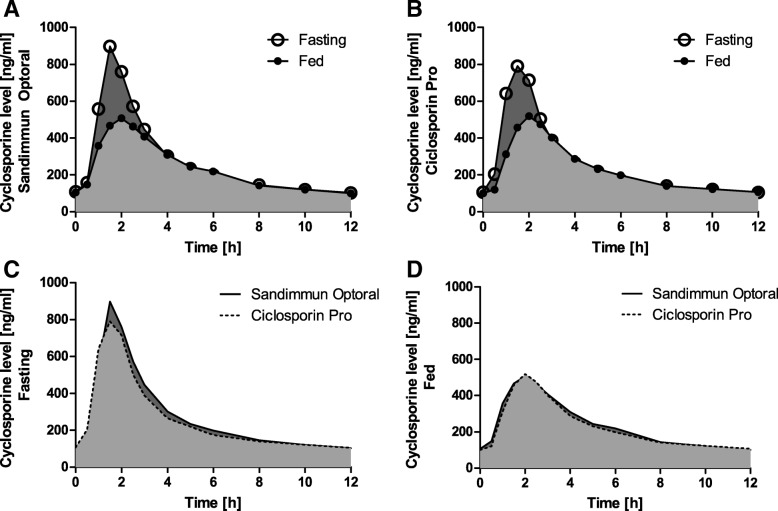
Pharmacokinetic profiles. Graphs show pharmacokinetic profiles of Sandimmun®Optoral (**a**) and Ciclosporin Pro (**b**) taken after 10 h of fasting or high-fat breakfast. Parts (**c**) and (**d**) illustrate the difference between Sandimmun®Optoral and Ciclosporin Pro in these conditions

The pharmacokinetic parameters AUC_SS, τ_, C_SS, max_, and C_SS, min_ measured under both nutrition conditions under Ciclosporin Pro as well as under Sandimmun® Optoral are displayed in Table 3 and Fig. 1.

**Table 3:** AUC_SS, τ_ [h*ng/ml], C_SS, max_ [ng/ml], and C_SS, min_ [ng/ml] for each treatment and nutrition condition (PP; *n* = 21)

	Ciclosporin Pro						Sandimmun Optoral					
	fasting			high-fat			fasting			high-fat		
	Geom. mean	95% CI	CV %	Geom. mean	95% CI	CV %	Geom. mean	95% CI	CV %	Geom. mean	95% CI	CV %
AUC	3006.5	[2652.7;3407.5]	28.0	2652.7	[2334.5;3014.2]	28.6	3228.6	[2857.8;3647.6]	27.3	2711.2	[2357.7;3117.7]	31.4
Cmax	844.6	[702.4;1015.7]	42.2	570.9	[463.6;703.2]	48.3	940.6	[802.7;1102.3]	35.9	560.2	[454.3;690.8]	48.6
Cmin	97.9	[87.1;110.1]	26.2	91.4	[81.1;103.0]	26.7	100.0	[88.8;112.5]	26.4	97.7	[86.9;109.9]	26.2

A difference in bioavailability due to high-fat nutrition of Ciclosporin Pro compared to Sandimmun® Optoral in terms of the difference D of ln-transformed bioavailability measured by the primary variables AUC_SS, τ_, C_SS, max_, and C_SS, min_ was not observed (Table 4). For the pharmacokinetic parameter C_SS, max_ the p-value of the one-sided t-test (α = 0.025) was 0.1820 (n = 21). The corresponding *p*-values for the other two parameters were > 0.025, respectively. Nevertheless, for the two pharmacokinetic parameters AUC_SS, τ_ and C_SS, max_ the arithmetic mean of the differences of ln-transformed nutrition effects between Ciclosporin Pro and Sandimmun® Optoral, i.e. D_CiclosporinPro_ – D_Sandimmun Optoral_ was slightly greater than zero, meaning that the reduction of bioavailability of cyclosporine caused by the ingestion of high-fat food immediately before medication intake appeared on average numerically less pronounced under Ciclosporin Pro than under Sandimmun® Optoral.

**Table 4:** Difference between Ciclosporin Pro and Sandimmun® Optoral in ln-transformed nutrition effects (PP, *n* = 21)

PK parameter	D_Ciclosporin Pro_ – D_Sandimmun Optoral_ Mean [SD]	D_Ciclosporin Pro_ – D_Sandimmun Optoral_ [97.5% CI]	Testing of H_0_^*^	
			t-value	p-value^**^
C_SS, max_	0.1266 [0.6248]	[−0.1578; Infinity]	0.93	0.1820
AUC_SS, τ_	0.0494 [0.2850]	[−0.0803; Infinity]	0.79	0.2180
C_SS, min_	−0.0464 [0.3271]	[−0.1953; Infinity]	− 0.65	0.7384

Bold entries are significant

* Testing of H_0_ (0 ≥ D_Ciclosporin Pro_ - D_Sandimmun Optoral_) by means of the one-sided paired t-test (α = 0.025)

** lowest (one-sided) significance level, for which the null hypothesis could be rejected

To explore the robustness of results, two analyses of variance (ANOVA) were calculated for each pharmacokinetic parameter by using a mixed model with a two-sided significance level of 5%, respectively. Both robustness analyses confirmed the results of the one-sided t-test (Additional file 6: Figure S6 and Additional file 7: Figure S7).

#### Nutrition effect on the bioavailability of Ciclosporin pro and Sandimmun® Optoral

The extent of the impact of nutrition on the bioavailability of Ciclosporin Pro and (separately) on the bioavailability of Sandimmun® Optoral was measured by the differences D_Ciclosporin Pro_ and D_Sandimmun Optoral_ (Table 5). According to the results of two-sided t-tests with a significance level of α = 0.05, for both study medications the null hypothesis, that there is no nutrition effect on the bioavailability of the respective medication can be rejected with respect to the pharmacokinetic parameters AUC_SS, τ_ and C_SS, max_ (two-sided *p*-values < 0.05). This means a nutrition effect was found for both study medications with respect to the parameters AUC_SS, τ_ and C_SS, max_, but not with respect to C_SS, min_.

**Table 5:** Difference of ln-transformed PK variables between high-fat versus fasting condition (PP; *n* = 21) for Ciclosporin Pro and Sandimmun® Optoral

PK parameter	D_Ciclosporin Pro_ Mean [SD]	D_Ciclosporin Pro_ [95% CI]	Testing of H_0_^*^	
			t-value	p-value^**^
AUC_SS, τ_	−0.1252 [0.2008]	[−0.2166; − 0.0338]	−2.86	**0.0097**
C_SS, max_	−0.3916 [0.4371]	[− 0.5906; − 0.1927]	−4.11	**0.0005**
C_SS, min_	− 0.0690 [0.2094]	[− 0.1644; 0.0263]	−1.51	0.1465
PK parameter	D_Sandimmun Optoral_ Mean [SD]	D_Sandimmun Optoral_ [95% CI]	Testing of H_0_^*^	
			t-value	p-value^**^
AUC_SS, τ_	−0.1747 [0.1443]	[−0.2403; − 0.1090]	−5.55	**<.0001**
C_SS, max_	−0.5183 [0.3148]	[− 0.6616; − 0.3750]	−7.55	**<.0001**
C_SS, min_	− 0.0227 [0.1789]	[− 0.1041; 0.0588]	−0.58	0.5681

* Testing of H_0_ (D_Ciclosporin Pro_ = 0) by means of the two-sided paired t-test

** lowest (two-sided) significance level, for which the null hypothesis could be rejected

#### Bioequivalence of Ciclosporin pro and Sandimmun® Optoral under fasting conditions

The bioequivalence of Ciclosporin Pro and Sandimmun® Optoral under fasting conditions and separately under fed conditions was explored by calculation of geometric means of the ratios of non-transformed pharmacokinetic variables including corresponding 90% confidence intervals (Table 6). The 90% confidence intervals based on AUC_SS, τ_ were within the acceptance interval [0.80;1.25] under fasting and high-fat conditions. However, with the exception of the PP set under high-fat condition, they were not within the more stringent acceptance interval [0.90; 1.11] for narrow therapeutic index drugs. Furthermore, the 90% confidence intervals based on C_SS, max_ were not included in any acceptance interval. Therefore, bioequivalence of Ciclosporin Pro and Sandimmun® Optoral (in the sense of similarity in AUC_SS, τ_ and C_SS, max_) – which had been proven in other studies - could neither be documented for fasting nor for high-fat conditions.

**Table 6:** Ratio of non-transformed PK variables of Ciclosporin Pro to Sandimmun® Optoral under fasting condition (top) and under high-fat conditions (bottom) (PP; *n* = 18)

	Bioavailability_Ciclo Pro_/Bioavailability_Optoral_ Geometric mean	Bioavailability_Ciclo Pro_/Bioavailability_Optoral_ [90% CI]
PK parameter (fasting)		
AUC_SS, τ_	0.940	[0.868; 1.018] ^a^
C_SS, max_	0.863	[0.745; 0.999]
C_SS, min_	1.011	[0.922; 1.108] ^b^
PK parameter (high-fat)		
AUC_SS, τ_	1.034	[0.972; 1.099] ^b^
C_SS, max_	1.082	[0.930; 1.260]
C_SS, min_	0.955	[0.881; 1.035] ^a^

^a^ CI included in [0.80; 1.25], but not in [0.90; 1.11]

^b^ CI included in [0.90; 1.11]

#### Safety evaluation

A total of 26 adverse events (AE) occurred in 14/31 patients (45.2%).

At first sight, the proportion of patients with AEs occurring under Ciclosporin Pro appeared higher than the proportion of patients with AEs occurring under Sandimmun® Optoral. However, in three patients bradycardia was documented as the only adverse event. Bradycardia was diagnosed based on the ECG at the final visit. Therefore, by definition the AEs were considered to have started under treatment with Ciclosporin Pro, although no ECG was performed after treatment with Sandimmun® Optoral and the lowest heart rate was already observed by Visit Day 29 (PK2) for all three patients. However, if the bradycardia events diagnosed using the ECG at the final visit are not counted as AEs with onset during Ciclosporin Pro, the number of individual events remains still slightly higher for the Ciclosporin Pro treatment period with no clear pattern.

There was only one adverse drug reaction (urinary tract infection, observed under treatment with Ciclosporin Pro) and no AE led to discontinuation or reduction of study drug. No death occurred during the study. Two serious adverse events were reported for two patients under treatment with Ciclosporin Pro (pancreatitis and relapse of ulcerative colitis). The causality was assessed as “no reasonable possibility” for both events as a relation to the study medication is medically extremely unlikely. Three AEs were related to laboratory parameters (increases in creatinine, gamma-glutamyltransferase and alkaline phosphatase). However, there was no laboratory parameter for which abnormalities were reported as an AE by more than one patient. Tolerability of both study drugs was assessed as very good or good by both the patients and investigators for all patients for whom an assessment was available.

## Discussion

Nutrition effects on bioavailability were found for the gel-based cyclosporine formulation Ciclosporin Pro as well as for the microemulsion Sandimmun® Optoral with respect to AUC_SS, τ_ and C_SS, max_. Effects were not captured by C_SS, min_. A significant difference in reduction of bioavailability due to high-fat nutrition between the two formulations could not be proven in the current trial (For *p*-values see Table 4).

Calcineurin inhibitors are immunosuppressive drugs with a relatively narrow therapeutic range. Overdosing results in acute toxicity not limited to the kidney, while underdosing is associated with allograft rejection [14]. Therapeutic drug monitoring is conventionally based on measurement of trough levels, although targeting C2 (the 2 h post-dose blood cyclosporine concentration) values might go along with reduced incidence of acute renal allograft rejection [15, 16]. High intra-individual variability of the second commonly used calcineurin inhibitor tacrolimus has been shown to go along with inferior allograft survival [17, 18] and development of de novo donor-specific antibodies [19]. Dietary compliance has been identified as a major problem in this setting [18]. Increased intra-individual variability of cyclosporine trough levels was associated with a higher incidence of acute rejection episodes, reduced long-term renal function and shortened graft survival [20]. Therefore, interventions to minimize the causes of high variability, not only of trough levels, but possibly on whole pharmacokinetic profiles, are essential to improve long-term outcomes following renal transplantation.

The influence of preprandial or postprandial administration on the pharmacokinetics of various cyclosporine formulations has been shown in various studies. Mueller et al. showed the influence of a high fat meal on cyclosporine bioavailability from the first marketed formulation (Sandimmun®) vs the microemulsion (Neoral®) in healthy volunteers. The food intake had minor influence on cyclosporine uptake from the microemulsion [5]. In contrast to our results, Kees et al. showed a higher AUC after a fat rich breakfast compared to the fasting AUC for a gel-based capsule formulation (Neoimmun®) in healthy volunteers. C_max_ of the fed and the fasting pharmacokinetic profiles were comparable with an increase after food intake in male subjects only [6]. Two Japanese studies in patients with psoriasis showed a consistently lower C_max_ when Neoral® (microemulsion) was taken postprandial compared to the preprandial administration (p not given and *p* < 0.05, respectively) [7, 8]. As administration after fasting is truly only possible in the morning and patient compliance concerning dietary restrictions is limited, a formulation which releases cyclosporine independently from food uptake going along with increased stability of pharmacokinetic profiles would be preferred.

Both cyclosporine formulations analysed in the current trial showed a nutrition effect resulting in a decreased AUC_SS, τ_ and C_SS, max_, while the usually recorded C_SS, min_ appeared unchanged reflecting the problem of trough level monitoring [21]. As administration after fasting twice daily is not practicable intra-individual differences in AUC_SS, τ_ and C_SS, max_ have to be assumed. This questions the whole concept of trough level monitoring. The development of a functional enzyme activity monitoring might be a future goal for optimizing therapy.

In the patient population analyzed, bioequivalence of Ciclosporin Pro and Sandimmun® Optoral (in the sense of similarity in both, AUC_SS, τ_ and C_SS, max_) could not be documented neither under fasting conditions nor under high-fat conditions. However, this study was not designed to evaluate bioequivalence. It has to be kept in mind that bioequivalence was assessed as an exploratory secondary endpoint and that the examined patient population for the evaluation of bioequivalence was not randomly selected but was a biased subset of patients who had stable C0 levels during the treatment phase, i.e. patients who required a change in the morning dose after switch of medication from Sandimmun® Optoral to Ciclosporin Pro were excluded from the bioequivalence evaluation.

### Strengths and limitations

Following sample size calculations, it was planned to enroll sufficient patients so that 30 patients would be available with four evaluable pharmacokinetic profiles. However, the study was stopped prematurely after 30 month and the enrolment of 31 patients due to recruitment difficulties within a reasonable time frame. The resulting number of evaluable patients, i.e. patients with four evaluable pharmacokinetic profiles was 24 in the FAS and 21 in the PP analysis set. This reduced the power of statistical testing.

This was an open-label study, thus the investigational team, the patient, and the clinical study monitor knew the sequence group during the study. Nevertheless, data management personnel and the statistician were unaware of the patient’s assignment to sequence group. The laboratory was blinded as well. The database was unblinded following completion of data cleaning, data quality control, and database lock. The allocation of patients to the per-protocol set was performed prior to unblinding, i.e. without knowledge of the patient’s sequence group. Therefore, influence on study results due to knowledge of assignment to study groups should be negligible.

Inclusion and exclusion criteria were defined with emphasis on patient safety. Therefore, as every change in immunosuppressive therapy is a risk factor for adverse events, especially stable transplant function was of interest. No changes in transplant function or rejection episodes in connection with the study were reported.

## Conclusions

High-fat food leads to changes in bioavailability of cyclosporine, not captured by trough level monitoring.

The reduction of bioavailability of cyclosporine caused by the ingestion of high-fat food immediately before medication intake was not different under Ciclosporin Pro compared to Sandimmun® Optoral. Conversion to Ciclosporin Pro seems to be safe with regard to intra-individual pharmacokinetic variability.

## Additional files

Additional file 1: **Figure S1.** Inclusion and exclusion criteria. (DOCX 27 kb)

Additional file 2: **Figure S2.** Diagram of study procedures. (DOCX 39 kb)

Additional file 3: **Figure S3.** Composition of breakfasts. (DOCX 32 kb)

Additional file 4: **Figure S4.** Concomitant medication during study. (DOCX 27 kb)

Additional file 5: **Figure S5.** Reasons for exclusion from the per-protocol analysis set. (DOCX 20 kb)

Additional file 6: **Figure S6.** ANOVA for ln-transformed nutrition effects (top) and Geometric Least Squares Means for nutrition effects and Ratios of Geometric Least Squares Means. (DOCX 28 kb)

Additional file 7: **Figure S7.** ANOVA for ln-transformed pharmacokinetic parameters (top) and Geometric Least Squares Means for pharmacokinetic parameters. (DOCX 32 kb)

## Abbreviations

AE: Adverse event
ANOVA: Analysis of variance
FAS: Full analysis set
GCP: Good clinical practices
GEM: Gel emulsion preconcentrate
HEENT: Head, eyes, ears, nose, throat
PK: Pharmacokinetic
PP: Per protocol analysis set
