# Supplementary material for: Randomized, open-label, comparative phase IV study on the bioavailability of Ciclosporin Pro (Teva) versus Sandimmun® Optoral (Novartis) under fasting versus fed conditions in patients with stable renal transplants
Source: BMC Nephrol. 2019 May 14;20:167. doi: 10.1186/s12882-019-1340-z (PMC6518767; doi:10.1186/s12882-019-1340-z)
Supplement: Supplementary file 1 — Figure S1. Inclusion and exclusion criteria. (DOCX 27 kb) [file 12882_2019_1340_MOESM1_ESM.docx]

Additional file 1: **Figure S1** Inclusion and exclusion criteria

| **INCLUSION CRITERIA** |
| --- |
| 1. Signed informed consent; |
| 1. Male or female, aged ≥ 18 years; |
| 1. One or two renal transplants and at least 6 months post-transplantation; |
| 1. Maintenance treatment with Sandimmun^®^ Optoral (with or without other immunosuppressive drugs), and regularly controlled by a transplantation center; |
| 1. Stable individualized dose of cyclosporine and no change in immunosuppressive regimen for at least 2 months prior to study start; |
| 1. Stable graft function for at least 2 months and < 20% change in creatinine clearance (MDRD) for at least 1 month prior to study start; |
| 1. Patient agreed to follow the study-specific instructions; |
| 1. Patient was able and willing (professional situation, family situation, planned holidays etc.) to come to the foreseen visits including four 13 hour-visits for the pharmacokinetic profiles. |
| **EXCLUSION CRITERIA** |
| 1. History of hypersensitivity to cyclosporine or any of the other excipients; |
| 1. Previous transplant with any organ other than kidney; |
| 1. Rejection episode within the past 6 months prior to the start of the study; |
| 1. Clinically relevant co-existing disease or other abnormal condition which might have compromised the function of gastrointestinal tract, kidney or liver or which could have influenced the cyclosporine pharmacokinetic profiles; |
| 1. Any active malignancy; |
| 1. Uncontrolled hypertension; |
| 1. Known or suspected significant hepatic impairment; |
| 1. Clinically significant hyperkalaemia (K^+^ > 6 mmol/l); |
| 1. Clinically significant laboratory and/or physical changes during the last 2 months prior to the start of the study; |
| 1. Change in concomitant medication (drugs known to affect the pharmacokinetics of cyclosporine) during the past 2 months prior to study start; |
| 1. Any use of drug, prescribed or over-the-counter, (except stable concomitant medication) within 2 weeks prior to the first administration of study medication except this will not affect the outcome of the study in the opinion of the clinical investigator; |
| 1. Pregnancy or breast feeding. Women of childbearing potential unable or unwilling to practice adequate contraceptive measures (i.e. oral contraceptive steroids, intrauterine device, sexual abstinence, vasectomized partner); |
| 1. Any other condition of the patient that in the opinion of the investigator may compromise evaluation of the study treatment or may jeopardize patient’s compliance or adherence to protocol requirements; |
| 1. Previous enrolment in this study or participation in any other drug investigational trial within the past 2 months (or five half-lives whatever is longer) prior to enrolment or simultaneous participation in another clinical trial. |
