# Supplementary material for: Randomized, open-label, comparative phase IV study on the bioavailability of Ciclosporin Pro (Teva) versus Sandimmun® Optoral (Novartis) under fasting versus fed conditions in patients with stable renal transplants
Source: BMC Nephrol. 2019 May 14;20:167. doi: 10.1186/s12882-019-1340-z (PMC6518767; doi:10.1186/s12882-019-1340-z)
Supplement: Supplementary file 2 — Figure S2. Diagram of study procedures. (DOCX 39 kb) [file 12882_2019_1340_MOESM2_ESM.docx]

Additional file 2: **Figure S2** Diagram of study procedures

|  | **ACTIVITIES** | **STUDY VISITS** | | | | | | |
| --- | --- | --- | --- | --- | --- | --- | --- | --- |
|  |  | **Visit 1 (Day -4 to 0)**  Sandimmun^®^ Optoral **Baseline** | **Visit 2 (D 7±3)**  Sandimmun^®^ Optoral **C0 level** | **Visit 3 (D 28±7)**  Sandimmun^®^ Optoral **PK1** | **Visit 4 (D 29±7)**  Sandimmun^®^ Optoral **PK2** | **Visit 5 (D 36±3)**  Ciclosporin Pro **C0 level** | **Visit 6  (D 57±7)**  Ciclosporin Pro **PK3** | **Visit 7  (D 58±7)**  Ciclosporin Pro **PK4 + Final visit** |
| **Procedures** | Patient information + written informed consent | • |  |  |  |  |  |  |
|  | Incl. + excl. criteria | • |  |  |  |  |  |  |
|  | Demographic data (incl. age, height) | • |  |  |  |  |  |  |
|  | Case history Incl. previous treatment | • |  |  |  |  |  |  |
|  | Vital signs + weight | • | • | • | • | • | • | • |
|  | Physical examination (incl. 12-lead ECG) | • |  |  |  |  |  | • |
|  | Randomisation | • |  |  |  |  |  |  |
| **Lab** | Urine pregnancy test | • |  |  |  |  |  |  |
|  | Urine analysis | • |  |  | • |  |  | • |
|  | Clinical Hematology + Serum Chemistry (incl. glomerular filtration rate) | • |  |  | • |  |  | • |
|  | Ciclosporin C0 levels | • | • | • | • | • | • | • |
|  | Pharmacokinetic profile |  |  | • | • |  | • | • |
| **Assessments** | Adverse Events |  | • | • | • | • | • | • |
|  | Concomitant medication | • | • | • | • | • | • | • |
|  | Adherence |  | • | • | • | • | • | • |
|  | Tolerability |  |  |  | • |  |  | • |
| **Drug** | Distribution/instructions | • | • |  | • | • |  | • |
|  | Return, check |  | • |  | • | • |  | • |
|  | Dose adjustment (if necessary) |  | • |  |  | • |  |  |
