# Supplementary material for: Randomized, open-label, comparative phase IV study on the bioavailability of Ciclosporin Pro (Teva) versus Sandimmun® Optoral (Novartis) under fasting versus fed conditions in patients with stable renal transplants
Source: BMC Nephrol. 2019 May 14;20:167. doi: 10.1186/s12882-019-1340-z (PMC6518767; doi:10.1186/s12882-019-1340-z)
Supplement: Supplementary file 3 — Figure S3. Composition of breakfasts. (DOCX 32 kb) [file 12882_2019_1340_MOESM3_ESM.docx]

Additional file 3: **Figure S3** Composition of breakfasts

| 1. **High-fat breakfast A** | | | | | | |
| --- | --- | --- | --- | --- | --- | --- |
|  |  | **kcal** | **proteins [g]** | **fat [g]** | **carbs [g]** |  |
| 60 g | whole-grain bread roll | 133 | 4.78 | 0.92 | 25.98 |  |
| 50 g | brown bread | 105 | 2.83 | 0.40 | 22.17 |  |
| 30 g | margarine | 217 | 0.08 | 24.00 | 0.15 |  |
| 20 g | gouda cheese, 40% fat | 60 | 4.94 | 4.46 | 0.00 |  |
| 45 g | camembert | 130 | 9.45 | 10.26 | 0.00 |  |
| 20 g | cream cheese | 67 | 2.20 | 6.30 | 0.50 |  |
| 20 g | strawberry jam | 54 | 0.06 | 0.03 | 13.01 |  |
|  | **Total** | **765** | **24.33**  **13%** | **46.37**  **55%** | **61.81**  **32%** |  |
|  | | | | | |  |
| 1. **High-fat breakfast B** | | | | | |  |
|  |  | **kcal** | **proteins [g]** | **fat [g]** | **carbs [g]** |  |
| 60 g | whole-grain bread roll | 133 | 4.78 | 0.92 | 25.98 |  |
| 50 g | brown bread | 105 | 2.83 | 0.40 | 22.17 |  |
| 30 g | margarine | 217 | 0.08 | 24.00 | 0.15 |  |
| 40 g | full-fat cheese, 50% fat | 138 | 8.40 | 11.51 | 0.00 |  |
| 40 g | poultry mortadella | 70 | 8.40 | 3.93 | 0.13 |  |
| 20 g | cream cheese | 67 | 2.20 | 6.30 | 0.50 |  |
|  | **Total** | **729** | **26.68**  **15%** | **47.06**  **58%** | **48.93**  **27%** |  |
|  | | | | | |  |
| 1. **High-fat breakfast C** | | | | | |  |
|  |  | **kcal** | **proteins [g]** | **fat [g]** | **carbs [g]** |  |
| 60 g | wheat roll | 163 | 4.98 | 1.14 | 33.30 |  |
| 60 g | whole-grain bread | 113 | 3.89 | 0.58 | 22.54 |  |
| 30 g | margarine | 217 | 0.08 | 24.00 | 0.15 |  |
| 20 g | Salami | 74 | 3.70 | 6.60 | 0.00 |  |
| 20 g | gouda cheese, 48% fat | 69 | 4.45 | 5.60 | 0.00 |  |
| 60 g | egg | 92 | 7.74 | 6.72 | 0.42 |  |
|  | **Total** | **728** | **24.93**  **14%** | **44.64**  **55%** | **56.41**  **31%** |  |
| 1. **Standard normal breakfast** | | | | | |  |
|  |  | **kcal** | **proteins [g]** | **fat [g]** | **carbs [g]** |  |
| 50 g | wheat roll | 137 | 4.53 | 0.95 | 27.75 |  |
| 20 g | butter | 148 | 0.13 | 16.64 | 0.12 |  |
| 20 g | jam (average) | 53 | 0.12 | 0.00 | 13.20 |  |
| 150 g | tea | - | 0.15 | 0.00 | 0.00 |  |
|  | **Total**  **Nutrition ratio** | **338** | **4.75**  **10%** | **17.59**  **30%** | **41.07**  **60%** |  |
