# Supplementary material for: Randomized, open-label, comparative phase IV study on the bioavailability of Ciclosporin Pro (Teva) versus Sandimmun® Optoral (Novartis) under fasting versus fed conditions in patients with stable renal transplants
Source: BMC Nephrol. 2019 May 14;20:167. doi: 10.1186/s12882-019-1340-z (PMC6518767; doi:10.1186/s12882-019-1340-z)
Supplement: Supplementary file 4 — Figure S4. Concomitant medication during study. (DOCX 27 kb) [file 12882_2019_1340_MOESM4_ESM.docx]

Additional file 4: **Figure S4** Concomitant medication during study. *F* Full analysis set, *P* per protocol analysis set.

| No. | Analysis set | Concomitant therapy |
| --- | --- | --- |
| 1 | FP | prednisone  amlodipine, allopurinol, valsartan, cinacalcet |
| 2 | FP | CellCept®  calcitriol, amoxicillin |
| 3 | FP | Myfortic®, prednisone  metoprolol, sodium bicarbonate, enalapril, amlodipine, ezetimib/simvastatin, phenprocoumon |
| 4 | FP | prednisone  metoprolol, ramipril, amlodipine, furosemide, sodium bicarbonate, cholecalciferol, iron (II) ion |
| 5 | FP | Myfortic®  fluvastatin, acetylsalicylic acid, allopurinol, candesartan, metoprolol, nitrendipine, cholecalciferol, calcipotriol |
| 6 | F | Myfortic®, prednisone  carvedilol, candesartan/hydrochlorothiazide |
| 7 | FP | prednisone, Imurek®  benzbromaron, opipramol, citalopram, pravastatin, omeprazole, acetylsalicylic acid |
| 8 | F | Myfortic®, prednisone  bisoprolol, amlodipine, pravastatin, omeprazole, sodium bicarbonate, cholecalciferol, enalapril, NaCl, ciprofloxacin, metronidazole, pethidine |
| 9 | FP | Myfortic®, prednisone  cholecalciferol, pravastatin, metoprolol, torasemide, pantoprazole, lamotrigine, valproic acid, lercanidipine, dihydralazine, repaglinide |
| 10 | FP | Myfortic®, prednisone  pantoprazole, amlodipine, bisoprolol, hydrochlorothiazide/triamteren, pravastatin |
| 11 | FP | prednisone  hydrochlorothiazide, fluvastatin, benzbromaron, calcium ion/cholecalciferol, nitrendipine |
| 12 | FP | Myfortic®, prednisone  calcitriol, erythropoetin |
| 13 | FP | prednisone, Imurek®  hydrochlorothiazide, alendronic acid, lovastatin, levo-thyroxin, nitrendipine |
| 14 | F | CellCept®, prednisone  bisoprolol, amlodipine, torasemide, alendronic acid, cholecalciferol |
| 15 | F | prednisone, Certican®  allopurinol, levo-thyroxin, bisoprolol, pantoprazole, acetylsalicylic acid, doxazosin, torasemide, lactulose, enalapril, pravastatin, lercanidipine, erythropoetine |
| 16 | FP | CellCept®, prednisone  acetylsalicylic acid, metoprolol, torasemide, moxonidine, ramipril, cholecalciferol, simvastatin, sodium bicarbonate, allopurinol |
| 17 | FP | Myfortic®, prednisone  enalapril, omeprazole, calcitriol |
| 18 | FP | Myfortic®, prednisone  cinacalcet, bisoprolol, acetylsalicylic acid, alendronic acid, pravastatin, candesartan, omeprazole, allopurinol, sodium bicarbonate, amlodipine, bisabolol, doxazosin, moxonidine |
| 19 | F | CellCept®, prednisone  nebivolol, candesartan plus, doxazosin |
| 20 | F | Sandimmun®, prednisone  magnesium, candesartan, phenprocoumon, pantoprazole, torasemide, benzbromaron, fluvastatin, sotalol, nitrendipine, moxonidine, colchicine |
| 21 | FP | prednisone, Imurek®  iron (II) ion, bisoprolol, amlodipine |
| 22 | FP | -  benzbromaron, bisoprolol, ramipril, acetylsalicylic acid, metamizole, hydrochlorothiazide |
| 23 | FP | prednisone  furosemide, benzbromaron, enalapril cor, pravastatin, nebivolol, amlodipine |
| 24 | FP | prednisone  acetylsalicylic acid, atenolol, nitrendipine, benzbromaron, cholecalciferol, ezetimib, tamsulosin |
| 25 | FP | prednisone, Certican®  amlodipine, bisoprolol, cholecalciferol, calcitriol, ramipril |
| 26 | F | prednisone  metoprolol, clonidine, acetylsalicylic acid, Alendron beta, torasemide, omeprazole, cinacalcet, amlodipine, dimenhydrinate, metronidazole, ciprofloxacin |
| 27 | FP | Myfortic®, prednisone  acetylsalicylic acid, lercanidipine, nebivolol, simvastatin, candesartan, omeprazole, furosemide, ezetimib/simvastatin, moxonidine, spironolactone, ezetimib, hydrochlorothiazide |
| 28 | F | Myfortic®, prednisone  torasemide, benzbromaron, ramipril, amlodipine, pravastatin, acetylsalicylic acid, cholecalciferol |
| 29 | F | Myfortic®, prednisone, Imurek®  cinacalcet, furosemide, omeprazole, metoprolol, ramipril, allopurinol, fluvastatin, mesalazine, ursodeoxycholic acid, cefuroxime, benzbromaron |
| 30 | F | prednisone  benzbromaron, pravastatin, nitrendipine, clonazepam, ramipril, roxithromycin |
| 31 | FP | Myfortic®, prednisone  levo-thyroxin, moxonidine, candesartan, furosemide, iron (II) ion, erythropoetine, leuproreline, magnesium, benzbromaron, lactulose, pravastatin, cyclocapronic acid, paracetamol, metoclopramide |
