# Supplementary material for: Randomized, open-label, comparative phase IV study on the bioavailability of Ciclosporin Pro (Teva) versus Sandimmun® Optoral (Novartis) under fasting versus fed conditions in patients with stable renal transplants
Source: BMC Nephrol. 2019 May 14;20:167. doi: 10.1186/s12882-019-1340-z (PMC6518767; doi:10.1186/s12882-019-1340-z)
Supplement: Supplementary file 5 — Figure S5. Reasons for exclusion from the per-protocol analysis set. (DOCX 20 kb) [file 12882_2019_1340_MOESM5_ESM.docx]

Additional file 5: **Figure S5** Reasons for exclusion from the per-protocol analysis set. A total of 10 patients were excluded. Thereof, 4 of 15 patients in Group A: fasting→fed and 6 of 16 patients (37.5%) in Group B: fed→fasting. Multiple reasons for exclusion were applicable in some patients.

|  | **Number of violations leading to exclusion from the PP analysis set** | | |
| --- | --- | --- | --- |
| **Reason for exclusion (violation classified as major)** | **fasting→fed** | **fed→fasting** | **Total** |
| Not four pharmacokinetic profiles evaluable | 2 | 5 | 7 |
| Diarrhoea or vomiting at the day of a PK profile | 1 | 0 | 1 |
| Not compliant concerning the timing of start of breakfast relative to the intake of study medication | 2 | 1 | 3 |
| Use of prohibited concomitant therapy | 0 | 1 | 1 |
| Other protocol violation* | 0 | 1 | 1 |

*patient received a high-fat instead of a normal breakfast
