# Supplementary material for: Randomized, open-label, comparative phase IV study on the bioavailability of Ciclosporin Pro (Teva) versus Sandimmun® Optoral (Novartis) under fasting versus fed conditions in patients with stable renal transplants
Source: BMC Nephrol. 2019 May 14;20:167. doi: 10.1186/s12882-019-1340-z (PMC6518767; doi:10.1186/s12882-019-1340-z)
Supplement: Supplementary file 6 — Figure S6. ANOVA for ln-transformed nutrition effects (top) and Geometric Least Squares Means for nutrition effects and Ratios of Geometric Least Squares Means. (DOCX 28 kb) [file 12882_2019_1340_MOESM6_ESM.docx]

Additional file 6: **Figure S6** ANOVA for ln-transformed nutrition effects (top) and Geometric Least Squares Means for nutrition effects and Ratios of Geometric Least Squares Means: Back-transformed from ANOVA using exponential function (bottom) (PP, n=21)

|  | **Test of fixed effect**  **Treatment** | | **Least Squares Means of Treatment** | | **Differences of Lsmeans**  **(Ciclosporin Pro – Sandimmun Optoral)** | | | |  |
| --- | --- | --- | --- | --- | --- | --- | --- | --- | --- |
|  | **F-value** | **p-value** | **Ciclosporin**  **Pro** | **Sandimmun Optoral** | **Estimate** | **t-value** | **p-value** | **95% CI** | |
| **D_Cmax_** | 1.15 | 0.2898 | -0.4387 | -0.5654 | 0.1266 | 1.07 | 0.2898 | [-0.1122;0.3654] | |
| **D_AUC_** | 0.85 | 0.3627 | -0.1512 | -0.2006 | 0.04944 | 0.92 | 0.3627 | [-0.05918;0.1581] | |
| **D_Cmin_** | 0.63 | 0.4313 | -0.09613 | -0.04975 | -0.04638 | -0.80 | 0.4313 | [-0.1644;0.07167] | |

|  | **Geometric Least Squares Means of Treatment** | | **Ratios of Geometric Lsmeans**  **(Ciclosporin Pro / Sandimmun Optoral)** | |
| --- | --- | --- | --- | --- |
|  | **Ciclosporin**  **Pro** | **Sandimmun Optoral** | **Estimate** | **95% CI** |
| **R_Cmax_** | 0.6449 | 0.5681 | 1.135 | [0.8939;1.4411] |
| **R_AUC_** | 0.8597 | 0.8182 | 1.051 | [0.9425;1.1713] |
| **R_Cmin_** | 0.9083 | 0.9515 | 0.955 | [0.8484;1.0743] |
