# Supplementary material for: Randomized, open-label, comparative phase IV study on the bioavailability of Ciclosporin Pro (Teva) versus Sandimmun® Optoral (Novartis) under fasting versus fed conditions in patients with stable renal transplants
Source: BMC Nephrol. 2019 May 14;20:167. doi: 10.1186/s12882-019-1340-z (PMC6518767; doi:10.1186/s12882-019-1340-z)
Supplement: Supplementary file 7 — Figure S7. ANOVA for ln-transformed pharmacokinetic parameters (top) and Geometric Least Squares Means for pharmacokinetic parameters. (DOCX 32 kb) [file 12882_2019_1340_MOESM7_ESM.docx]

Additional file 7: **Figure S7** ANOVA for ln-transformed pharmacokinetic parameters (top) and Geometric Least Squares Means for pharmacokinetic parameters: Back-transformed from ANOVA using exponential function (bottom) (PP, n=21)

| **PK parameter** | **Test of fixed effect**  **Treatment*Nutr.Condition** | |  | **Least Squares Means of**  **Treatment*Nutr. Condition** | |
| --- | --- | --- | --- | --- | --- |
|  | **F-value** | **p-value** | **Treatment,**  **Nutr. Condition** | **Estimate** | **95% CI** |
| **lnC_SS,max_** | 1.24 | 0.2707 | CiclosporinPro,fed | 6.3717 | [6.1537;6.5896] |
|  |  |  | CiclosporinPro,fasting | 6.7633 | [6.5454;6.9812] |
|  |  |  | SandimmunOptoral,fed | 6.3526 | [6.1347;6.5706] |
|  |  |  | SandimmunOptoral,fasting | 6.8709 | [6.6530;7.0888] |
| **lnAUC_SS,τ_** | 0.59 | 0.4459 | CiclosporinPro,fed | 7.8882 | [7.7368;8.0397] |
|  |  |  | CiclosporinPro,fasting | 8.0135 | [7.8620;8.1649] |
|  |  |  | SandimmunOptoral,fed | 7.9101 | [7.7586;8.0615] |
|  |  |  | SandimmunOptoral,fasting | 8.0847 | [7.9333;8.2362] |
| **lnC_SS,min_** | 0.42 | 0.5182 | CiclosporinPro,fed | 4.5083 | [4.3729;4.6438] |
|  |  |  | CiclosporinPro,fasting | 4.5774 | [4.4419;4.7128] |
|  |  |  | SandimmunOptoral,fed | 4.5755 | [4.4401;4.7110] |
|  |  |  | SandimmunOptoral,fasting | 4.5982 | [4.4627;4.7337] |

| **PK parameter** |  | **Least Squares Means of**  **Treatment*Nutr. Condition** | |
| --- | --- | --- | --- |
|  | **Treatment,**  **Nutr. Condition** | **Estimate** | **95% CI** |
| **C_SS,max_** | CiclosporinPro,fed | 585.05 | [470.455;727.490] |
|  | CiclosporinPro,fasting | 865.49 | [696.035;1076.209] |
|  | SandimmunOptoral,fed | 573.98 | [461.601;713.798] |
|  | SandimmunOptoral,fasting | 963.82 | [775.106;1198.469] |
| **AUC_SS,τ_** | CiclosporinPro,fed | 2665.64 | [2291.129;3101.683] |
|  | CiclosporinPro,fasting | 3021.47 | [2596.709;3515.370] |
|  | SandimmunOptoral,fed | 2724.66 | [2341.624;3170.042] |
|  | SandimmunOptoral,fasting | 3244.45 | [2788.614;3775.167] |
| **C_SS,min_** | CiclosporinPro,fed | 90.77 | [79.273;103.939] |
|  | CiclosporinPro,fasting | 97.26 | [84.936;111.364] |
|  | SandimmunOptoral,fed | 97.08 | [84.783;111.163] |
|  | SandimmunOptoral,fasting | 99.31 | [86.721;113.716] |
